# Dynamics and Protective Effectiveness of Serological Testing Among Healthcare Workers Vaccinated Against COVID-19

**DOI:** 10.3390/medicina62050810

**Published:** 2026-04-24

**Authors:** Vilija Gurkšnienė, Tadas Alčauskas, Dovilė Karosienė, Jurgita Urbonienė, Fausta Majauskaitė, Mindaugas Paulauskas, Birutė Zablockienė, Dalius Vitkus, Ligita Jančorienė

**Affiliations:** 1 Faculty of Medicine, Vilnius University, 03101 Vilnius, Lithuania; vilija.gurksniene@santa.lt (V.G.); fausta.majauskaite@santa.lt (F.M.); mindaugas.paulauskas@santa.lt (M.P.); birute.zablockiene@santa.lt (B.Z.); dalius.vitkus@santa.lt (D.V.); ligita.jancoriene@santa.lt (L.J.); 2 Vilnius University Hospital Santaros Clinics, 08410 Vilnius, Lithuania; dovile.karosiene@santa.lt (D.K.); jurgita.urboniene@santa.lt (J.U.)

**Keywords:** antibodies, COVID-19, healthcare workers, vaccination

## Abstract

*Background and Objectives*: Healthcare workers are at heightened risk of SARS-CoV-2 infection. Understanding the duration and protective value of vaccine-induced immunity is critical to inform booster strategies. This study investigates longitudinal dynamics of anti-SARS-CoV-2 receptor-binding domain IgG (anti-RBD IgG) antibodies and their association with infection risk among vaccinated healthcare workers. *Materials and Methods*: A prospective cohort study was conducted at Vilnius University Hospital Santaros Klinikos, Lithuania. A total of 1778 healthcare workers who completed a primary COVID-19 vaccination series were followed. Blood samples were collected every three months to measure anti-RBD IgG levels. Participants also received up to three booster doses. COVID-19 was identified by PCR, antigen tests, or positive anti-nucleocapsid IgG. For serologically detected cases, infection timing was assigned to the interval between study visits. Antibody dynamics were analyzed across vaccination stages, time, age groups, and circulating SARS-CoV-2 variants. *Results*: Anti-RBD IgG titers peaked in the first quarter after primary vaccination (mean 7904 AU/mL), declined sharply by quarters 2–3, and rose substantially after booster doses. Following the first booster, titers increased to ~12,598 AU/mL in quarter 1 and continued rising through quarter 3. The highest levels were observed after the second booster (24,456 AU/mL in Q1), followed by gradual decline. A high-titer plateau persisted from quarters 6 to 9 (~21,000 AU/mL), followed by decline in quarters 10–11 and partial rebound in Q12. Approximately 49.6% of participants experienced COVID-19 during follow-up. Antibody response patterns were similar across age groups, with only minor transient differences. *Conclusions*: COVID-19 booster doses significantly enhance and prolong humoral immunity in healthcare workers compared with the primary vaccination series. However, antibody waning over time emphasizes the need for timely boosters, particularly during periods of variant circulation. These findings support continued booster vaccination and monitoring of long-term immune protection, although anti-RBD IgG should be interpreted as a surrogate marker of humoral rather than overall immunity.

## 1. Introduction

In December 2019, the new SARS-CoV-2 virus, which causes COVID-19, was detected in Hubei Province, China, and has affected the entire world. According to data from the World Health Organization (WHO), as of 14 September 2025, there have been nearly 779 million cases of COVID-19 and approximately 7.1 million deaths worldwide [1]. Healthcare workers are among those most at risk of contracting SARS-CoV-2 [2,3]. A study of serological data from 2063 healthcare workers in the United Kingdom found that medical workers are three times more likely to contract COVID-19 than the general population [4]. According to research data, one of the groups of medical workers most at risk of infection is those working in emergency medical departments (31–33% of all infected medical workers) [5,6]. A particularly high risk of contracting COVID-19 has been identified among general practice nurses. According to various sources, this professional group accounts for 48–55% of healthcare professionals infected in hospitals [7,8].

One of the main goals in combating the new global pandemic was to develop an effective vaccine against COVID-19. The first vaccines were developed a year after the SARS-CoV-2 virus was identified. One of the first vaccines was produced by scientists working on a program coordinated by Pfizer and BioNTech. The genetic material chosen as the basis for this vaccine was messenger ribonucleic acid (mRNA). During clinical trials phases I and II, it was observed that one of the vaccine versions tested in the program, codenamed BNT162b2, elicited an adequate immune response [9]. Further studies have shown that two doses of the BNT162b2 vaccine provide 95% protection against COVID-19 in people over 16 years of age [10]. On 21 December 2020, the European Commission granted PfizerBioNTech conditional approval to market the COMIRNATY^®^ (BNT162b2) vaccine for the immunization of individuals over 16 years of age against the SARS-CoV-2 virus [11].

In September 2020, the WHO Strategic Advisory Group of Experts on Immunization prepared guidelines for vaccination against COVID-19, stating that healthcare professionals should be among the priority groups [12]. On 23 December 2020, the Minister of Health of the Republic of Lithuania issued an order establishing priority groups for COVID-19 vaccination, which also gave priority to healthcare workers (Order No. V-3006) [13]. With the start of the immunization of medical workers against COVID-19 infection, the effectiveness of vaccination in this priority group is also being analyzed. A study of 23,324 healthcare workers in the United Kingdom found that the incidence of COVID-19 in the unvaccinated group was 14 cases per 10,000 person-days worked, while in the group of workers who received the second dose of the BNT162b2 mRNA vaccine, the rate was only 4 cases per 10,000 person-days [14]. Studies conducted in Tel Aviv, Israel, and Boston, United States, also showed a significantly lower number of COVID-19 after vaccination [15,16].

However, even with good immunization results among healthcare workers, there remains a small risk of contracting COVID-19 or becoming a carrier of the SARS-CoV-2 virus. At Israel’s largest healthcare facility, Sheba Medical Center, 39 cases of infection were detected among 1497 fully vaccinated employees tested using the PCR method. Thirty-three percent of these cases were asymptomatic carriers, while 67% had a mild form of the disease. All infected individuals had lower titers of antibodies against the SARS-CoV-2 virus in serological tests compared to groups of uninfected employees [17].

In August 2021, the United States Centers for Disease Control and Prevention (CDC) emphasized the danger of the Delta variant of the SARS-CoV-2 virus. According to the CDC, the Delta variant spreads twice as fast as previous variants of the virus [18]. According to a study conducted in the United Kingdom, the BNT162b2 mRNA vaccine is 88% effective against the Delta SARS-CoV-2 variant and 93.7% effective against the Alpha variant [19].

Since COVID-19 vaccination has only recently begun, new virus subtypes are constantly emerging, making it very important to monitor and analyze the effectiveness of immunization in vaccinated individuals. This is particularly relevant for healthcare workers who work with immunocompromised patients or to ensure the safety of medical staff working on the front lines of the fight against the COVID-19 pandemic.

The aim of our study is to evaluate the antibody titer dynamics in healthcare workers who have been vaccinated with the COVID-19 vaccine according to the full vaccination schedule and who have been vaccinated with a booster dose of the COVID-19 vaccine, and to assess the incidence of COVID-19 among healthcare workers who have been vaccinated with the COVID-19 vaccine.

## 2. Materials and Methods

A prospective observational cohort study was conducted at Vilnius University Hospital Santaros Klinikos (VUH SK), Vilnius, Lithuania. Participants included VUH SK employees (all of the employees were healthcare workers) who had completed a primary COVID-19 vaccination series and provided informed consent. The primary vaccination series was defined as either two doses of BNT162b2 (Pfizer-BioNTech/Comirnaty; Pfizer Inc., New York, NY, USA; BioNTech Manufacturing GmbH, Mainz, Germany) or mRNA-1273 (Spikevax; ModernaTX, Inc., Cambridge, MA, USA) vaccine (administered within an 8-week interval), a single dose of the Janssen vaccine, or a confirmed SARS-CoV-2 infection following one vaccine dose.

Blood samples were collected every three months to measure receptor-binding domain-specific IgG antibodies (anti-RBD IgG). The SARS-CoV-2 IgG II Quant assay (Abbott, Chicago, IL, USA) a chemiluminescent microparticle immunoassay (CMIA) was used for the quantitative determination of IgG antibodies to SARS-CoV-2 in human serum and plasma on the ARCHITECT ci8200 System. This assay is an automated, two-step immunoassay for the quantitative determination of IgG antibodies to SARS-CoV-2 in human serum and plasma using chemiluminescent microparticle immunoassay (CMIA) technology. Sample, SARS-CoV-2 antigen coated paramagnetic microparticles, and assay diluent are combined and incubated. The IgG antibodies to SARS-CoV-2 present in the sample bind to the SARS-CoV-2 antigen coated microparticles. The mixture is washed. Anti-human IgG acridinium-labeled conjugate is added to create a reaction mixture and incubated. Following a wash cycle, Pre-Trigger and Trigger Solutions are added. The resulting chemiluminescent reaction is measured as a relative light unit (RLU). There is a direct relationship between the amount of IgG antibodies to SARS-CoV-2 in the sample and the RLU detected by the system optics.

COVID-19 cases were identified by positive PCR or rapid antigen tests, or a positive anti-nucleocapsid IgG (anti-N IgG) result (≥1.4 S/C). For participants diagnosed by PCR or antigen testing, the date of the first positive test was used as the infection date. For participants identified only by anti-N IgG seroconversion, infection was assigned to the interval between the last anti-N-negative and the first anti-N-positive study visit; accordingly, the timing of these infections should be interpreted as approximate.

SARS-CoV-2 testing by PCR was performed on the Cepheid GeneXpert Dx GX-XVI platform using the Xpert Xpress CoV-2 plus assay. This cartridge-based real-time RT-PCR assay qualitatively detects SARS-CoV-2 RNA by targeting the N, E, and RdRP regions of the viral genome. Specimens (nasopharyngeal swabs) were processed according to the manufacturer’s instructions by transferring the collected swab specimen medium into the assay cartridge, which was then inserted into the GeneXpert instrument (Cepheid, Sunnyvale, CA, USA) for fully automated nucleic acid extraction, amplification, and detection. Internal sample processing and probe check controls were included in each run, and results were reported using the GeneXpert software (version 4.7b).

For rapid antigen testing samples were analyzed with the CerTest SARS-CoV-2 + Flu A + Flu B + Respiratory syncytial virus (RSV) combo card test (CerTest Biotec, S.L., San Mateo de Gállego, Zaragoza, Spain), a rapid lateral-flow immunochromatographic assay for qualitative detection of SARS-CoV-2, influenza A, influenza B, and RSV nucleoprotein antigens from nasopharyngeal swabs. Specimen processing and interpretation were performed according to the manufacturer’s instructions, with results read at 10 min.

Statistical analyses were performed at both the descriptive and inferential levels and focused on quarter-level summaries of longitudinal serological kinetics. Anti-RBD IgG concentrations are presented as mean ± standard deviation with 95% confidence intervals, and categorical variables as number (%). Because repeated measurements were obtained from the same participants over time, longitudinal comparisons were evaluated using linear mixed-effects models with participant-level random intercepts to account for within-subject correlation. Fixed effects included quarter, vaccination stage, and age group, with interaction terms examined where relevant. Pairwise comparisons between quarters, vaccination stages, and age groups were derived from the fitted mixed-effects models, and *p* values were adjusted for multiple comparisons using the Bonferroni method. All tests were two-sided, and an adjusted *p* value < 0.05 was considered statistically significant. The course of the COVID-19 pandemic in Lithuania was marked by distinct periods of SARS-CoV-2 variant dominance: the Beta variant from 1 October 2020–1 July 2021; the Delta variant until 31 December 2021; the Omicron variant until 31 July 2022; and subsequently, the Omicron XBB subvariant.

The design of the study was approved by Vilnius Regional Biomedical Research Ethics Committee, Permission to conduct biomedical research No. 2021/11-1395-870.

## 3. Results

This study analyzed data from 1778 participants (mean age 46.51 ± 12.7 years); 84.5% were female. Of the 1778 participants, 1471 (82.7%) received one booster dose, 180 (10.1%) received two booster doses, and 3 (0.2%) received three booster doses. The first booster dose was administered a mean of 240.02 ± 26.46 days after completion of the primary vaccination series; the second booster dose, 770.32 ± 206.86 days after completion of the primary series. Participant characteristics are presented in Table 1.

In total, 15,327 blood samples were collected for antibody measurement: 1834 prior to completion of the primary vaccination series, 4346 after completion of the primary series, 8864 after the first booster dose, 278 after the second booster dose, and 5 after the third booster dose.

**Figure 1:**
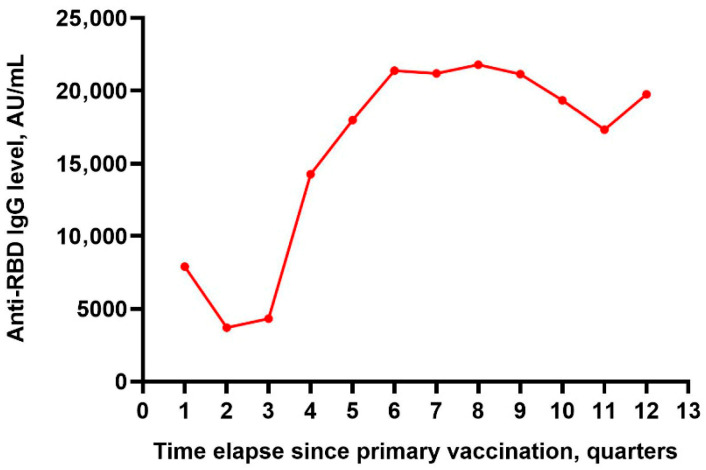
Longitudinal anti-RBD IgG antibody dynamics.

In the first quarter (Q1) after completion of the primary vaccination series, anti-RBD IgG averaged 7904.55 ± 9541.42 AU/mL. Levels then fell significantly in Q2 to 3704.86 ± 5645.59 AU/mL (*p* < 0.001 vs. Q1) and remained low in Q3 (4314.02 ± 8589.86 AU/mL; *p* < 0.001 vs. Q1). In Q4, titers rose sharply to 14,247.56 ± 12,381.86 AU/mL (*p* < 0.001 vs. Q1–Q3), increased further in Q5 to 17,984.15 ± 14,320.81 AU/mL (*p* < 0.001 vs. Q1–Q4), and peaked in Q6 at 21,379.20 ± 14,924.45 AU/mL (*p* < 0.001 vs. Q1–Q5). Across the high plateau (Q6–Q9), differences in anti-RBD IgG levels were not significant. Titers then declined in Q10 (19,341.11 ± 12,047.08 AU/mL; *p* < 0.001 vs. Q6–Q9) and Q11 (17,317.06 ± 11,256.68 AU/mL; *p* < 0.001 vs. Q6–Q10), followed by a partial rebound in Q12 (19,744.48 ± 12,267.78 AU/mL) (Table 2, Figure 1).

**Figure 2:**
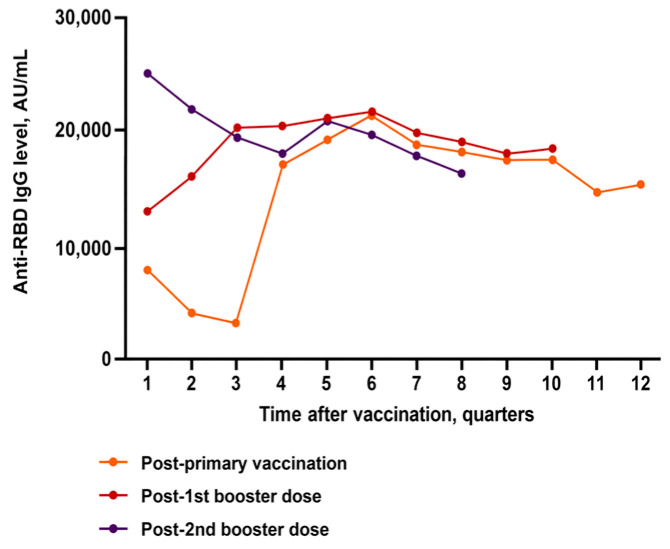
Longitudinal anti-RBD IgG antibody titers after primary vaccination and booster doses.

Anti-RBD IgG titers were substantially higher after booster doses than after the primary vaccination series, and were higher after the second booster compared with the first booster in the first two post-vaccination quarters. In Q1, anti-RBD IgG titers were 7904.55 ± 9541.42 AU/mL after the primary series, 12,597.91 ± 12,316.80 AU/mL after the first booster, and 24,456.50 ± 16,263.73 AU/mL after the second booster (all pairwise *p* < 0.001). Q2 showed the similar pattern (3687.57 ± 5628.21 vs. 15,649.72 ± 13,313.79 vs. 21,400.63 ± 13,022.02 AU/mL respectively; all *p* < 0.001). In Q3, anti-RBD IgG titers after both boosters remained higher than after the primary series, while titers after the first and second boosters did not differ significantly. In Q4, the difference in anti-RBD IgG titers between the primary series and the first booster was small but significant (17,632.20 ± 13,302.44 vs. 20,975.77 ± 14,887.02 AU/mL; *p* = 0.029); titers after the primary series and the second booster did not differ, nor did titers after the first and second boosters. From Q5 to Q10, no statistically significant differences in anti-RBD titers were detected between dose groups (Table 3, Figure 2).

Over the first year following vaccination, distinct response patterns were observed across the primary vaccination series, the first booster, and the second booster. In Q1 after completion of the primary series, anti-RBD IgG averaged 7904.55 ± 9541.42 AU/mL, then fell significantly in Q2 to 3687.57 ± 5628.21 AU/mL (*p* < 0.001 vs. Q1) and reached the lowest point in Q3 (3067.94 ± 6912.43 AU/mL; *p* < 0.001 vs. Q1 and vs. Q2). In Q4, titers rose sharply to 17,632.20 ± 13,302.44 AU/mL.

A similar pattern was observed after the second booster. In Q1 after the second booster, anti-RBD IgG averaged 24,456.50 ± 16,263.73 AU/mL, then fell in Q2 to 21,400.63 ± 13,022.02 AU/mL (*p* = 0.001 vs. Q1), in Q3 to 19,354.01 ± 12,275.08 AU/mL (*p* = 0.032 vs. Q1), and reached the lowest point in Q4 (18,215.76 ± 11,939.41 AU/mL; *p* < 0.001 vs. Q1).

After the first booster, a different pattern was observed: after reaching 12,597.91 ± 12,316.80 AU/mL in Q1, anti-RBD IgG titers increased to 15,649.72 ± 13,313.79 AU/mL in Q2 (*p* = 0.225 vs. Q1) and further to 20,989.82 ± 15,095.80 AU/mL in Q3 (*p* = 0.032 vs. Q1), remaining high in Q4 (Figure 2).

**Figure 3:**
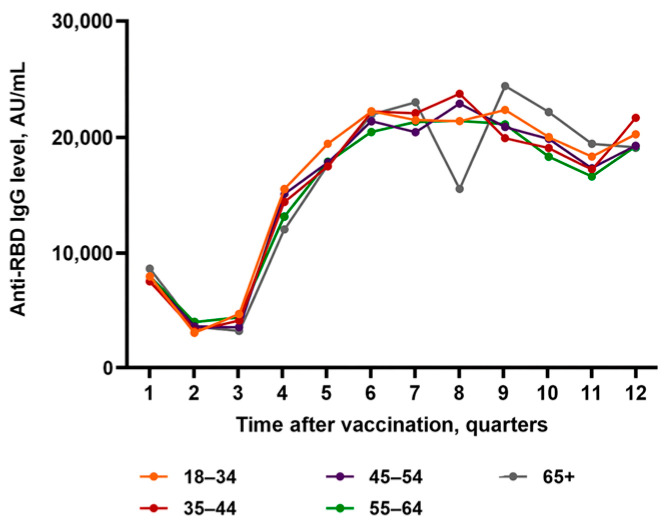
Longitudinal anti-RBD IgG antibody dynamics across age groups.

Some between-group differences were observed in selected quarters. In Q2, the youngest group (18–34 years) had significantly lower titers than the 55–64-year group (3244.42 ± 4730.20 vs. 4171.05 ± 6808.28 AU/mL; *p* = 0.020). In Q4, the youngest group showed significantly higher titers than the 55–64-year group (15,369.74 ± 13,555.26 vs. 13,016.96 ± 11,598.90 AU/mL; *p* = 0.044). In Q8, the 65+-year group had the lowest values, significantly below the 35–44- and 45–54-year groups (15,390.05 ± 12,202.14 vs. 23,582.04 ± 16,162.31 and 22,755.17 ± 15,891.70 AU/mL; *p* = 0.021 and *p* = 0.031, respectively). In Q9, the anti-RBD IgG in 35–44-year group was lower than in 18–34-year and 65+-year groups (19,769.29 ± 12,474.39 vs. 22,195.63 ± 12,694.99 and 24,261.65 ± 12,923.28 AU/mL; *p* = 0.048 and *p* = 0.023). In Q10, the 65+-year group had the lowest titers (18,980.69 ± 11,891.82 AU/mL), significantly below the 55–64-year group (19,124.07 ± 12,942.62 AU/mL; *p* = 0.049). Overall, we observed no consistent age-related differences in the longitudinal dynamics of anti-RBD IgG across age groups (Table 4, Figure 3).

## 4. Discussion

In this prospective cohort of vaccinated healthcare workers, anti-RBD IgG titers showed a clear dose- and time-dependent pattern. After completion of the primary vaccination series, antibody levels were highest in the first post-vaccination quarter and then declined markedly over the following two quarters, whereas booster doses induced a substantially stronger response and maintained higher titers for longer. These findings are consistent with the broader literature, which shows that vaccine-induced humoral immunity is strongest during the first weeks to three months after primary immunization and then progressively wanes over time [20,21,22,23,24,25,26,27]. Although anti-RBD IgG is not a direct measure of virus neutralization, anti-RBD levels generally correlate with neutralizing activity and can therefore serve as a useful population-level surrogate of humoral protection; however, this relationship is imperfect and may be attenuated by immune escape in newer variants [26].

The pronounced decline of anti-RBD IgG concentration observed after the primary series in our cohort is biologically plausible and agrees with prior longitudinal studies. Anastassopoulou et al. [20] and Oliveira-Silva et al. [21] both reported a steep fall in anti-RBD/anti-spike IgG within three months of BNT162b2 vaccination, while Bayart et al. [23], Dakovic Rode et al. [24], and Krintus et al. [25] documented continued waning by six months. Taken together with our data, these studies support the interpretation that the primary vaccine series generates a robust early antibody response but does not sustain peak humoral protection indefinitely, especially in populations with ongoing occupational exposure to SARS-CoV-2. This pattern is in line with the general observation from the literature that declining antibody titers over time are an expected feature of post-vaccination immunity rather than an isolated finding [20,21,22,23,24,25,26,27,28,29,30,31,32,33,34,35,36,37,38,39].

An important finding of our study is the marked rise in anti-RBD IgG after booster vaccination. In the first quarter after the first booster, titers exceeded those seen after primary vaccination, and the second booster induced an even stronger early response. This agrees with studies that showed that booster doses consistently re-amplify humoral immunity in healthcare workers [40,41,42,43,44,45,46,47,48,49,50,51,52,53,54,55,56,57,58,59,60,61]. Grassi et al. [52] reported a pronounced increase in IgG shortly after boosting, followed by a slower decline over subsequent months, while Augustinussen et al. [55] and Guibert et al. [45] showed that booster doses help restore or equalize humoral responses across different primary vaccination regimens. Our results therefore reinforce the concept that revaccination remains an effective strategy for prolonging serological protection in high-risk occupational groups.

Notably, in our cohort the first booster was followed by a sustained high plateau of anti-RBD IgG concentration and even further increase from Q1 to Q3, whereas the second booster produced the highest early titers of anti-RBD IgG but was followed by a gradual decline. This difference may reflect the real-world conditions in which healthcare workers acquired immunity, including varying intervals between vaccine doses, repeated antigenic stimulation, and possible exposure to circulating SARS-CoV-2 variants during follow-up. Similar heterogeneity in post-booster kinetics of anti-RBD IgG concentration has been described in previous studies, where prior infection and repeated immune stimulation were associated with stronger or more persistent serologic responses to vaccination against COVID-19 [40,41,42,53,60]. Because our cohort was followed across periods dominated by different viral variants, the antibody trajectories observed here likely reflect combined vaccine-induced and hybrid immune stimulation rather than vaccine timing alone. In addition, because a proportion of infections were identified by anti-N IgG seroconversion rather than virological testing, the exact timing of some infections could only be approximated, which should be considered when interpreting temporal links between infection and subsequent antibody changes.

With respect to age, we observed only isolated quarter-specific differences and no consistent long-term age-related pattern in anti-RBD IgG dynamics. This partially contrasts with many studies from the literature review, in which younger healthcare workers often had higher titers after primary vaccination or after boosters [20,21,26,31,38,52]. At the same time, not all published findings were uniform: some studies did not identify significant age effects after adjustment or found that age-related differences diminished over time [22,30,33]. Our results suggest that in a highly vaccinated healthcare-worker population receiving booster doses, the effect of age on longitudinal humoral responses may be attenuated, although the lower titers observed in the oldest participants in selected later quarters indicate that immunosenescence may still contribute in some subgroups.

From a clinical and public-health perspective, our findings are particularly relevant for healthcare workers, who remain at increased occupational risk of SARS-CoV-2 exposure and onward transmission [62,63,64,65]. Previous reports have shown that vaccinated healthcare workers with lower antibody titers may still develop breakthrough infection, and the literature further emphasized that waning humoral immunity after primary vaccination raises the question of optimal revaccination intervals [40,41,42,43,44,45,46,47,48,49,50,51,52,53,54,55,56,57,58,59,60,61]. In this context, the substantially higher titers of anti-RBD IgG observed after booster doses in our study support continued booster strategies for healthcare personnel, especially during periods of intense viral circulation or when caring for vulnerable patient populations. At the same time, our data also show that antibody decline continues even after boosting, underscoring the need for ongoing surveillance rather than assuming durable protection after any single dose.

Humoral markers nevertheless capture only one component of post-vaccination im-munity. Cellular immune responses, particularly memory T-cell responses, may contribute to protection against severe disease even when circulating antibody titers decline. Because cellular immunity was not measured in this study, the durability of overall immune protection may be greater than suggested by anti-RBD IgG kinetics alone.

## 5. Strengths and Limitations

This study has several strengths, including the large cohort size, repeated serological sampling, and prolonged follow-up across multiple post-vaccination quarters. These features allowed us to compare humoral responses after primary vaccination, the first booster, and the second booster within the same healthcare setting with ongoing real-world exposure. Nevertheless, several limitations should be acknowledged. First, the cohort was predominantly composed of female healthcare workers (84.5%), which may limit the generalizability of the findings to more sex-balanced healthcare worker populations. Second, the 65+ age group was underrepresented (5.1%), which may have reduced the precision of age-specific estimates and limited the interpretation of anti-body dynamics in older healthcare workers. Second, anti-RBD IgG was used as a marker of humoral response, but neutralizing activity and cellular immunity were not assessed; therefore, serological titers should not be interpreted as a complete measure of protection. Third, infections identified through anti-N IgG provided evidence of incident infection during follow-up, but their timing was less precise than for PCR- or antigen-confirmed cases. In addition, as in other observational studies of healthcare workers [26,28,29,40,41,42,66], prior infection, undocumented asymptomatic exposure, variant circulation, and the timing of booster uptake may have influenced antibody kinetics. Despite these limitations, the consistency between our results and previously published longitudinal studies strengthens the conclusion that booster doses substantially enhance, but do not permanently stabilize, humoral immunity against SARS-CoV-2 in healthcare workers.

## 6. Conclusions

This prospective cohort study demonstrated that anti-RBD IgG antibody levels among vaccinated healthcare personnel exhibit dynamic and dose-dependent patterns over time. After completion of the primary vaccination series, antibody titers peaked in the first quarter and declined significantly over the following two quarters, indicating waning immunity. Booster vaccination effectively restored and significantly enhanced antibody levels, with the highest titers observed after the second booster dose.

Following booster administration, anti-RBD IgG titers rose sharply and stabilized at a high plateau (quarters 6–9), remaining substantially higher than post-primary vaccination levels during the same timeframes. Although a gradual decline was observed from quarters 10–11, a modest rebound occurred in quarter 12, suggesting continued immune stimulation from either boosters or natural exposure to circulating variants.

Antibody responses were broadly consistent across age groups, with only isolated and transient differences observed in specific quarters, indicating that age had no sustained effect on long-term humoral response dynamics.

Overall, this study confirms that booster doses significantly enhance and prolong humoral immunity compared to the primary vaccination course. However, antibody waning remains evident over time, emphasizing the importance of timely booster administration to maintain humoral responses, especially in healthcare personnel with continued exposure risk. These findings support ongoing booster strategies and underscore the need for further research into neutralizing capacity, cellular immunity, hybrid immunity, and protection against emerging SARS-CoV-2 variants such as Omicron XBB.

## Figures and Tables

**Table 1:** The characteristics of participants, N (%).

Characteristic	Participants	Anti-RBD IgG Measurements
Sex		
Male	276 (15.5)	2383 (15.5)
Female	1502 (84.5)	12,944 (84.5)
Age group		
18–34	418 (23.5)	3599 (23.5)
35–44	316 (17.8)	2739 (17.9)
45–54	485 (27.3)	4221 (27.5)
55–64	469 (26.4)	4002 (26.1)
65+	90 (5.1)	766 (5.0)
Number of booster doses		
No	124 (7.0)	4346 (28.4)
One	1471 (82.7)	8864 (57.8)
Two	180 (10.1)	278 (1.8)
Three	3 (0.2)	5 (0.0)
COVID-19 during the study period	882 (49.6)	7640 (49.9)
Prevalent SARS-CoV-2 variant	At participant entry	At time of sample collection
Beta	1718 (96.6)	3780 (24.7)
Delta	58 (2.0)	2076 (13.5)
Omicron	2 (0.2)	4193 (27.4)
Omicron XBB	0	5278 (34.4)

**Table 2:** Longitudinal anti-RBD IgG antibody dynamics.

Quarter	N	Anti-RBD IgG	95% CI	Statistically Significant Difference from Quarters
Quarter 1	1027	7904.55 ± 9541.42	7320.31–8488.78	2, 3, 4, 5, 6, 7, 8, 9, 10, 11, 12
Quarter 2	1622	3704.86 ± 5645.59	3429.91–3979.82	1, 4, 5, 6, 7, 8, 9, 10, 11, 12
Quarter 3	1311	4314.02 ± 8589.86	3848.61–4779.43	1, 4, 5, 6, 7, 8, 9, 10, 11, 12
Quarter 4	901	14,247.56 ± 12,381.86	13,437.98–15,057.13	1, 2, 3, 5, 6, 7, 8, 9, 10, 11, 12
Quarter 5	2026	17,984.15 ± 14,320.81	17,360.20–18,608.11	1, 2, 3, 4, 6, 7, 8, 9, 10, 12
Quarter 6	1378	21,379.20 ± 14,924.45	20,590.51–22,167.88	1, 2, 3, 4, 5, 10, 11
Quarter 7	1348	21,182.02 ± 14,543.27	20,404.96–21,959.08	1, 2, 3, 4, 5, 10, 11
Quarter 8	447	21,791.93 ± 14,728.49	20,422.83–23,161.02	1, 2, 3, 4, 5, 10, 11, 12
Quarter 9	1115	21,143.48 ± 12,908.66	20,384.97–21,901.99	1, 2, 3, 4, 5, 10, 11
Quarter 10	1024	19,341.11 ± 12,047.08	18,602.36–20,079.85	1, 2, 3, 4, 5, 6, 7, 8, 9, 11
Quarter 11	1069	17,317.06 ± 11,256.68	16,641.51–17,992.62	1, 2, 3, 4, 6, 7, 8, 9, 10, 12
Quarter 12	225	19,744.48 ± 12,267.78	18,132.81–21,356.15	1, 2, 3, 4, 5, 8, 11

**Table 3:** Longitudinal anti-RBD IgG antibody titers after primary vaccination and booster doses.

Quarter	After Primary Vaccination	After 1st Booster Dose	After 2nd Booster Dose	p1	p2	p3
Quarter 1	7904.55 ± 9541.42	12,597.91 ± 12,316.80	24,456.50 ± 16,263.73	<0.001	<0.001	<0.001
Quarter 2	3687.57 ± 5628.21	15,649.72 ± 13,313.79	21,400.63 ± 13,022.02	<0.001	<0.001	<0.001
Quarter 3	3067.94 ± 6912.43	20,989.82 ± 15,095.80	19,354.01 ± 12,275.08	<0.001	<0.001	0.296
Quarter 4	17,632.20 ± 13,302.44	20,975.77 ± 14,887.02	18,215.76 ± 11,939.41	0.029	0.802	0.137
Quarter 5	19,481.27 ± 14,495.18	21,670.98 ± 14,505.65	21,555.02 ± 12,856.01	0.129	0.592	0.975
Quarter 6	21,338.99 ± 13,126.00	21,451.95 ± 13,041.75	19,734.84 ± 10,229.42	0.938	0.725	0.694
Quarter 7	18,628.08 ± 11,836.31	19,679.34 ± 12,416.70	18,146.74 ± 12,876.38	0.476	0.933	0.782
Quarter 8	18,818.55 ± 12,587.98	18,400.74 ± 11,790.29	16,294.64 ± 3964.76	0.800	0.648	0.691
Quarter 9	17,779.82 ± 11,853.83	17,539.18 ± 11,374.21		0.865		
Quarter 10	17,671.83 ± 10,442.61	18,597.87 ± 13,690.21		0.787		
Quarter 11	14,974.00 ± 10,239.86					
Quarter 12	15,452.08 ± 10,449.37					

p1: primary vs. 1st booster; p2: primary vs. 2nd booster; p3: 1st vs. 2nd booster.

**Table 4:** Longitudinal anti-RBD IgG antibody dynamics across age groups.

Quarter	18–34	35–44	45–54	55–64	65+
Quarter 1	7993.20 ± 10,023.90	7557.41 ± 9365.77	7853.33 ± 9524.30	7967.12 ± 9281.86	8618.96 ± 9397.89
Quarter 2	3244.42 ± 4730.20	3455.78 ± 4497.37	3803.05 ± 5890.98	4171.05 ± 6808.28	3828.02 ± 5178.44
Quarter 3	4880.20 ± 9024.75	4296.85 ± 8793.12	3731.53 ± 7599.12	4579.84 ± 9132.39	3386.79 ± 7726.83
Quarter 4	15,369.74 ± 13,555.26	14,230.33 ± 11,707.80	14,902.10 ± 12,657.46	13,016.96 ± 11,598.90	11,916.98 ± 11,026.74
Quarter 5	19,270.57 ± 14,697.02	17,304.79 ± 14,014.77	17,665.95 ± 14,176.72	17,751.47 ± 14,358.04	17,441.77 ± 14,169.54
Quarter 6	22,049.88 ± 14,401.13	22,053.84 ± 15,475.93	21,238.78 ± 14,912.17	20,318.74 ± 14,869.88	21,747.48 ± 15,692.89
Quarter 7	21,330.34 ± 13,690.21	21,924.20 ± 17,059.05	20,287.91 ± 13,941.17	21,155.88 ± 14,271.21	22,864.03 ± 13,299.66
Quarter 8	21,256.58 ± 12,267.90	23,582.04 ± 16,162.31	22,755.17 ± 15,891.70	21,271.78 ± 14,740.93	15,390.05 ± 12,202.14
Quarter 9	22,195.63 ± 12,694.99	19,769.29 ± 12,474.39	20,731.36 ± 12,944.49	20,987.87 ± 13,251.68	24,261.65 ± 12,923.28
Quarter 10	19,913.78 ± 11,382.01	18,945.66 ± 12,000.51	19,724.91 ± 12,567.65	18,207.88 ± 11,932.39	22,002.65 ± 12,577.17
Quarter 11	18,169.92 ± 10,842.69	17,043.45 ± 11,508.84	17,201.03 ± 11,320.80	16,499.43 ± 11,301.72	19,298.15 ± 11,466.50
Quarter 12	20,154.62 ± 12,048.56	21,512.31 ± 12,638.54	19,184.87 ± 11,916.22	19,124.07 ± 12,942.62	18,980.69 ± 11,891.82

## Data Availability

The original contributions presented in this study are included in the article. Further inquiries can be directed to the corresponding author.
